# Appendicitis in De Garengeot's Hernia Presenting as a Nontender Inguinal Mass: Case Report and Review of the Literature

**DOI:** 10.1155/2014/932638

**Published:** 2014-03-04

**Authors:** K. Ahmed, K. Bashar, T. J. McHugh, S. M. McHugh, E. Kavanagh

**Affiliations:** ^1^Department of Surgery, University Hospital Limerick, Dooradoyle, Limerick, Ireland; ^2^School of Medicine, Trinity College Dublin, Dublin 2, Ireland; ^3^Department of Surgery, Royal College of Surgeons in Ireland, Dublin 2, Ireland

## Abstract

*Introduction*. De Garengeot first described a femoral hernia containing the appendix in 1731. Appendicitis occurring in this rare clinical setting represents a challenge in diagnosis and management. *Case Presentation*. We present the case of a 71-year-old male with a three-day history of a nontender inguinal mass. Computed tomography (CT) suggested a femoral hernia. Intraoperatively, the hernia sac was noted to contain a perforated appendix. *Discussion*. This is an infrequently reported clinical scenario and only the 14th reported case in peer-reviewed literature which includes preoperative CT images. Our case adds to previously reported low sensitivity of CT for diagnosing De Garengeot's hernia. Furthermore, unlike our case the vast majority of previous reports noted a painful inguinal swelling. *Conclusion*. Perforated appendicitis in a femoral hernia is an extremely uncommon presentation. However, consideration should be given to De Garengeot's hernia in patients with a groin mass, even if nontender.

## 1. Introduction

De Garengeot's hernia is named after the 18th century Parisian surgeon Rene Jacques Croissant De Garengeot (1688–1759) [1]. This hernia was first described in 1731 and describes a femoral hernia including the appendix within the hernia sac. This is a rare presentation with the appendix present in only 0.8% of femoral hernias [1]. Appendicitis occurring in this rare clinical setting represents a challenge in diagnosis and management. To the authors' knowledge, there have been only 13 reported cases in published peer-reviewed literature with preoperative computed tomography (CT) imaging. Nine of these cases were included in one comprehensive review [2].

## 2. Case Report

**Figure 1:**
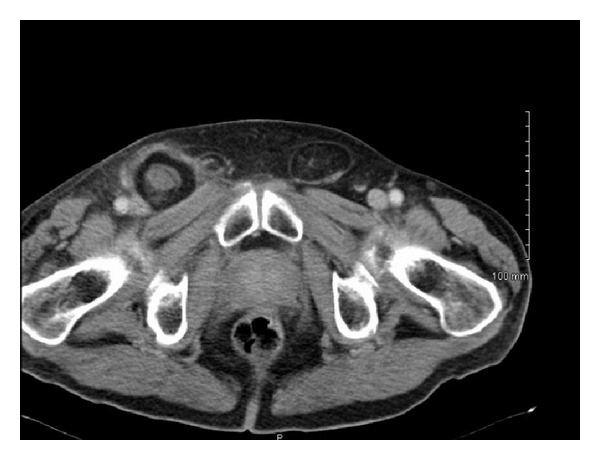
Preoperative CT scan demonstrating incarcerated femoral hernia containing a perforated appendix.

A 71-year-old male was referred by his general practitioner to a tertiary hospital surgical assessment unit with query right groin lymphadenopathy. The patient reported a three-day history of painless swelling in his right groin. Physical exam revealed a firm irreducible nontender mass in his right groin. There was no cough impulse present. His abdominal exam was otherwise soft and nontender. The patient was afebrile, normotensive and was not tachycardic with a heart rate of 98. Serological investigations revealed that leukocyte count was 11.4 × 10^9^/L. Renal and liver function tests were within normal limits. The provisional diagnosis was one of right inguinal lymphadenopathy. Computed tomography (CT) imaging was arranged (Figure 1). The CT report described a right femoral hernia containing omentum. The patient was then scheduled for emergency incarcerated femoral hernia repair.

A low approach for repair of femoral hernia was undertaken at surgery. Intraoperatively, the hernia sac was found to be containing a perforated appendix. A lower midline incision was additionally performed to allow reduction of the hernia sac back into the abdominal cavity and subsequent appendicectomy. The hernia defect was repaired using 0 PDS through the low approach incision. Postoperatively, the patient developed a superficial surgical site infection (SSI) in the lower half of the midline laparotomy wound. This was treated successfully with intravenous antibiotics. The patient was discharged home well two weeks after procedure for followup in outpatients. Subsequent histological examination of the appendix confirmed acute perforated appendicitis.

## 3. Discussion

In the United States alone there are an estimated 27,000 cases of femoral hernia annually, accounting for 3% of all hernias. The appendix is noted as within the femoral hernia sac in less than 1% of these [3]. Furthermore an even smaller proportion may be found with appendicitis, perforation, or necrosis, as in this case. Our case details the clinical difficulties in the diagnosis and management of this rare presentation of acute appendicitis. Furthermore, it is only the 14th case reported wherein preprocedural CT imaging is available.

Our case presented some interesting diagnostic challenges. The patient did not report any abdominal pain, and furthermore on examination the groin mass was not tender as might be clinically expected. A previous case series of 36 patients with De Garengeot's hernia noted the presence of a right groin mass in 97% of cases. However, the vast majority of these (97%) were painful, with only one patient presenting with a nontender mass as was the case with our patient [2].

The majority of patients presenting with De Garengeot's hernia are brought directly to surgery with the diagnosis of incarcerated femoral hernia made based upon clinical exam [3, 4].

Computed tomography findings of De Garengeot's hernia classically demonstrate intramural air density in an incarcerated hernia which indicates intestinal involvement but lacks obstruction of the small bowel [5, 6]. In the previously referenced comprehensive review preoperative CT scanning was obtained in 9 patients; however, in only 44% was the CT diagnostic for De Garengeot's hernia [2]. Our case report would add further weight to the suggestion that CT sensitivity is low in the diagnosis of appendicitis within a femoral hernia. Magnetic resonance imaging (MRI) has been suggested as alternative imaging tool to be considered [7].

Previous alternative management options have included incision and drainage with delayed appendectomy [8]. In our case, management of appendicitis within a femoral hernia sac involved appendicectomy with subsequent close of the femoral hernia defect which is more commonly reported. We utilised a PDS repair of the femoral defect. Although the hernia sac containing the perforated appendix and the hernia itself was intact and reduced into the abdominal cavity, some authors have previously suggested the avoidance of synthetic mesh due to the increased risk of SSI [3, 9]. Given the relative paucity of reported cases available in published literature there is no consensus on whether mesh can be used where there is little contamination of the surgical field. What has been previously noted is a significantly higher SSI rate overall in this rare cohort of patients when compared with similar patients with intraperitoneal perforation of the appendix, with authors noting a fivefold increase in infection rates [10]. Similarly, in our patient his postoperative stay was complicated by a wound infection of his midline laparotomy wound treated successfully with intravenous antibiotics without contamination of the low approach incision site.

## 4. Conclusion

Perforated appendicitis in a femoral hernia sac is an extremely uncommon presentation. However consideration should be given to De Garengeot's hernia in patients with a groin mass, even if nontender. This is only the 14th reported case with preoperative CT imaging. Preoperative imaging may be beneficial in diagnosing a femoral hernia; however, sensitivity for CT diagnosis of appendicitis within the hernia would appear to be low.
